# Resveratrol‐induced remodelling of myocellular lipid stores: A study in metabolically compromised humans

**DOI:** 10.14814/phy2.14692

**Published:** 2021-01-21

**Authors:** Nynke van Polanen, Evelyn Zacharewicz, Marlies de Ligt, Silvie Timmers, Esther Moonen‐Kornips, Gert Schaart, Joris Hoeks, Patrick Schrauwen, Matthijs K. C. Hesselink

**Affiliations:** ^1^ Department of Nutrition and Movement Sciences NUTRIM School for Nutrition and Translational Research in Metabolism Maastricht University Medical Centre+ Maastricht The Netherlands; ^2^ Department of Animal Sciences Division of Human and Animal Physiology Wageningen University Wageningen The Netherlands

**Keywords:** intramyocellular

## Abstract

In non‐athletes, insulin sensitivity correlates negatively with intramyocellular lipid (IMCL) content. In athletes, however, a pattern of benign IMCL storage exists, which is characterized by lipid storage in type I muscle fibres, in small and numerous lipid droplets (LDs) preferable coated with PLIN5, without affecting insulin sensitivity. Administration of resveratrol has been promoted for its beneficial effects on glucose homeostasis. We observed that 30 days of oral resveratrol administration (150 mg/day) in metabolically compromised individuals showed a 33% increase in IMCL (placebo vs. resveratrol; 0.86 ± 0.090 AU vs. 1.14 ± 0.11 AU, *p* = 0.003) without impeding insulin sensitivity. Thus, the aim of the present study was to examine if a resveratrol‐mediated increase in IMCL content, in metabolically compromised individuals, changes the LD phenotype towards the phenotype we previously observed in athletes. For this, we studied IMCL, LD number, LD size, subcellular distribution and PLIN5 coating in different fibre types using high‐resolution confocal microscopy. As proof of concept, we observed a 2.3‐fold increase (*p* = 0.038) in lipid accumulation after 48 h of resveratrol incubation in cultured human primary muscle cells. In vivo analysis showed that resveratrol‐induced increase in IMCL is predominantly in type I muscle fibres (placebo vs. resveratrol; 0.97 ± 0.16% vs. 1.26 ± 0.09%; *p* = 0.030) in both the subsarcolemmal (*p* = 0.016) and intermyofibrillar region (*p* = 0.026) and particularly in PLIN5‐coated LDs (*p* = 0.024). These data indicate that administration of resveratrol augments IMCL content in metabolically compromised individuals towards a LD phenotype that mimics an ‘athlete like phenotype’.

## Graphical abstract

Resveratrol increases IMCL in type I muscle fibers originating primarily from an increase in number of lipid droplets rather than size.

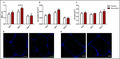

## 1 INTRODUCTION

Compromised insulin‐mediated glucose uptake in muscle is commonly observed in middle‐aged, overweight individuals and is a hallmark in the pathogenesis of insulin resistance and type 2 diabetes mellitus (T2DM) development (DeFronzo & Tripathy, 2009). In sedentary populations, there is a negative association between insulin sensitivity and intramyocellular lipid (IMCL) storage (Goodpaster et al., 2000; Krssak et al., 1999; Pan et al., 1997). Paradoxically, trained athletes with similarly high IMCL levels as patients with T2DM are highly insulin sensitive (Goodpaster et al., 2001). This paradox appears to originate from differences in lipid composition (Metcalfe et al., 2019), lipid morphology (Daemen et al., 2018; Nielsen et al., 2017) and/or subcellular distribution of lipid droplets (LDs) within the muscle (Daemen et al., 2018; Perreault et al., 2018).

To alleviate insulin resistance and its metabolic consequences, caloric restriction and physical activity are among the most powerful interventions. However, long‐term adherence to these interventions is low (Middleton et al., 2013). This has triggered the search for drug or nutritional interventions that mimic the effects of caloric restriction and/or exercise. In this respect, the polyphenol resveratrol is of interest as it promotes mitochondrial function and metabolic health in model systems via SIRT1 and PGC1α (Lagouge et al., 2006). In rodents, resveratrol blunted high‐fat diet‐induced hepatic lipid storage (Alberdi et al., 2013; Zhou et al., 2018), muscle lipid storage (Chen et al., 2011; Gong et al., 2020; Zhang et al., 2019) and improved insulin sensitivity (Jeon et al., 2012; Sun et al., 2007). Improved glucose and lipid handling was also observed in some human trials (Bhatt et al., 2012; Brasnyo et al., 2011; Timmers et al., 2011), but not in others (de Ligt et al., 2018; Kjaer et al., 2017; Poulsen et al., 2013; Timmers et al., 2016; van der Made et al., 2015; Yoshino et al., 2012). The inconsistency in these results may originate from differences in dosing, type of resveratrol used and phenotypical differences in the target population. Administration of 150 mg transresveratrol daily for 30 days was performed in three populations; middle aged normoglycaemic obese individuals (Timmers et al., 2011), middle‐aged overweight first‐degree relatives of patients with T2DM with compromised glucose homeostasis (de Ligt et al., 2018) and patients with T2DM (Timmers et al., 2016). In these studies, resveratrol administration consistently improved mitochondrial function while the effects on markers of insulin sensitivity were less consistent. Interestingly, the improvement in mitochondrial function was paralleled by increased muscle lipid and reduced liver lipid content in two of these studies (Timmers et al., 2011, 2016), effects similar to what has been observed after exercise training.

In highly insulin‐sensitive trained athletes, IMCL is stored predominantly in type I muscle fibres and dispersed alongside myofibrils (intermyofibrillar (IMF) LDs) in numerous small LDs (Daemen et al., 2018), with significantly more PLIN5 present in athletes than in T2DM individuals, a LD phenotype we referred to as an ‘athlete like phenotype’ (Gemmink et al., 2020). PLIN5 is a LD coat protein known to promote mitochondrial biogenesis and function (Bosma et al., 2013), in a SIRT1 and PGC1α‐dependent fashion (Najt et al., 2020). Upon exercise training, the morphological LD characteristics in patients with T2DM changed towards an athlete‐like phenotype, along with improved insulin sensitivity (Daemen et al., 2018). These observations prompted us to examine the hypothesis that in metabolically compromised individuals, a resveratrol‐mediated increase in IMCL content changes the LD phenotype from the phenotype observed in metabolically compromised individuals towards the phenotype we observed in (highly insulin‐sensitive) trained individuals.

To examine if the potency of resveratrol to promote muscle lipid storage is a cell autonomous effect, we first examined the effect of resveratrol in an in vitro system of cultured primary muscle cells. Subsequently, we examined if resveratrol‐induced elevation of IMCL after 30 days of in vivo resveratrol supplementation changed the morphological characteristics of myocellular LDs such that they mimic the profile of lipid storage previously observed in athletes.

## 2 METHODS

### 2.1 Ethical approval

The current study relies on a retrospective analyses of samples obtained from three different clinical trials registered as NCT00998504, NCT01638780 and NCT02129595 (de Ligt et al., 2018; Timmers et al., 2011, 2016) as well as on primary myotubes grown from previously obtained muscle biopsies (Vosselman et al., 2015). The Medical Ethic Committee of Maastricht University approved the original studies in accordance with the Declaration of Helsinki. All participants provided written informed consent before participation.

### 2.2 Design of the in vivo study

During the in vivo part of the study, individuals received 150 mg/day trans‐resveratrol (99.9%) and a placebo for 30 days in a double‐blinded, crossover trial with a washout period of at least 30 days. Middle‐aged, obese, normoglycaemic individuals (OB *n* = 11) (Timmers et al., 2011), individuals with an increased risk of developing diabetes for having at least one first‐degree relative with T2DM along with impaired glucose homeostasis (FDR *n* = 13) (de Ligt et al., 2018) or patients with type 2 diabetes (T2DM *n* = 17) (Timmers et al., 2016) were studied. All participants were men. Details of these studies can be found in the respective papers. At baseline, body mass, BMI and body fat (DXA) were determined. After 27–30 days of supplementation (placebo or resveratrol), fasting blood plasma values of glucose and insulin (to compute HOMA‐IR (Matthews et al., 1985)), free fatty acids (FFA) and triglycerides (TG) and plasma values of resveratrol and its direct metabolite dihydroresveratrol (DHR) were measured. A graded exercise test on a stationary bike was performed to assess maximal oxygen uptake (VO_2_max). Basal fatty acid oxidation was determined from indirect calorimetry. Ex vivo mitochondrial function was measured in permeabilized muscle fibres with high‐resolution respirometry (OROBOROS Instruments, Innsbruck, Austria) by measuring state 3 respiration (ADP stimulated respiration after addition of malate and octanoylcarnitine) and state U (maximal uncoupled respiration induced by titration of FCCP).

### 2.3 Muscle biopsies and sample subgroup selection

Muscle biopsies were obtained from the m. *vastus lateralis* in the morning at day 30 according to the Bergstrom method (Bergstrom, 1975) and processed for further analysis. Quantitative histological examination of IMCL by Oil red O was performed in 88% (36 of 41) of the individuals. In the remaining 12%, the histological appearance of the biopsy was insufficient to permit valid quantitative IMCL analysis. IMCL content (lipid area within a muscle fibre/area of the muscle fibre*100%) along with muscle fibre typing (immunolabelling of myosin heavy‐chain type I) was quantified essentially according to Koopman et al. (2001). Sections from both conditions (placebo and resveratrol) were randomly mounted on the same glass slides, imaged and analysed in a blinded fashion. For all three studies, putative ‘batch‐effects’ in the histological staining were accounted for by adjusting the data of the individual studies to the mean of that study. This resulted in a dataset with 36 individuals, showing a significant increase in IMCL content of 33% upon resveratrol administration (0.86 ± 0.09 AU in placebo vs. 1.14 ± 0.11 AU upon resveratrol, *p* = 0.003, Table 1).

**TABEL 1:** Subject characteristics of the complete group and subgroup for immunohistochemistry

	All participants (*n* = 36)		Subgroup (*n* = lO)		*p*‐value*
	953770‐21844000Placebo	Resveratrol	Placebo	Resveratrol	
Age (y)	61 ± 8		58 ± 8		0.415
BMI (kg/m^2^)	30.2 ± 2.6		30.5 ± 2.8		0.810
Body fat (%)	26.7 ± 3.6		27.2 ± 3.0		0.685
Resveratrol (ng/ml)	ND	356.0 ± 201.2	ND	337.4 ± 195.5	
DHR (ng/ml)	ND	415.0 ± 359.7	ND	323.3 ± 281.5	
Glucose (mmol/L)	6.3 ± 1.3	6.3 ± 1.4	6.2 ± 0.9	6.2 ± 1.0	0.847
Insulin (pmol/L)	82.7 ± 35.5	82.2 ± 35.0	94.1 ± 56.6	92.3 ± 47.1	0.458
HOMA‐IR	3.3 ± 1.3	3.3 ± 1.6	3.6 ± 1.9	3.6 ± 1.7	0.519
TG (mmol/L)	1.93 ± 1.13	1.97 ± 0.93	2.34 ± 1.73	2.38 ± 1.29	0.369
FFA (mmol/L)	0.72 ± 0.27	0.75 ± 0.25	0.78 ± 0.23	0.87 ± 0.23	0.528
IMCL (AU)	0.86 ± 0.54	1.14 ± 0.64^†^	0.73 ± 0.34	1.44 ± 0.74^†^	0.492
V02 max (ml/kg per min)	25.3 ± 4.8	25.0 ± 5.3	26.8 ± 4.3	27.1 ± 5.5	0.375
FAO (µmol/kg BW/min)	3.3 ± 0.8	3.2 ± 0.9	3.1 ± 0.9	3.0 ± 0.9	0.640
State3 (pmol/(s*mg))	27.9 ± 5.9	31.5 ± 7.1^†^	29.2 ± 6.9	34.0 ± 8.7^†^	0.561
StateU (pmol/(s*mg))	84.2 ± 19.3	93.3 ± 20.1^†^	83.7 ± 18.8	100.4 ± 16.6^†^	0.944

Data are expressed as mean ± SD.

BMI, body mass index; DHR, dihydroresveratrol; FAO, fatty acid oxidation, State3, ADP stimulated mitochondrial respiration after addition of malate and octanoylcarnitine; FFA, free fatty acids; HOMA‐IR, Homeostatic Model Assessment for Insulin Resistance; IMCL, intramyocellular lipids; ND, non detectable; TG, triacylglycerol; VO_2_max, maximal aerobic capacity; StateU, maximal uncoupled respiration induced by titration of FCCP.

* *p* < 0.05; values are significant different between resveratrol and placebo (Student's paired *t*‐test).

^†^ *p*‐values reflect the difference between all participants (*n* = 36) versus the subgroup (*n* = 10) in the placebo arm (Student's unpaired *t*‐test).

Given the extremely laborious nature of the detailed analysis of LD morphology, subcellular distribution and coating with relevant proteins, we studied our hypothesis in a subset of the original study population. Since the complete group consists of OB, FDR and T2DM individuals, we selected 3–4 individuals from each study that displayed an increase in IMCL (primary inclusion criteria). Secondary inclusion factors were high‐quality histology (essential for valid confocal microscopy) and a match for age, BMI and VO_2_max with the complete group. This resulted in 10 individuals (3 OB, 3 FDR and 4 T2DM) whose biopsies were processed for detailed analysis of LD characteristics in the present study. The subgroup is a valid reflection of the complete group as indicated by the similar subject characteristics (Table 1).

### 2.4 Cell culturing

Satellite cells were previously isolated, as described by Sparks et al. (2011), from muscle biopsies of three untrained individuals (Vosselman et al., 2015). Primary human myoblasts were cultured in growth medium containing low‐glucose (5.5 mM) Dulbecco's Modified Eagle Medium (DMEM) (Gibco, Thermo Fisher Scientific), 10% foetal bovine serum (Gibco), 1% fetuin (Sigma‐Aldrich), 0.7% bovine serum albumin (Sigma‐Aldrich), 0.1% dexamethasone (Sigma‐Aldrich), 0.1% Gentamycin (Gibco), 0.1% human epidermal growth factor (Gibco) and 0.02% Fungizone (Gibco). At 70–80% confluence, medium was replaced by a differentiation medium containing low‐glucose DMEM, 2% horse serum (Gibco), 1% fetuin and 2% penicillin and streptomycin (Sigma‐Aldrich). Cells were maintained at 37°C and 5% CO_2_ and medium was replaced every 2–3 days. After 6 days of differentiation, myotubes were incubated with or without 50 ng/ml resveratrol (R‐5010, Sigma‐Aldrich) for 48 h, inspired by work from others (Kim et al., 2013).

### 2.5 Live cell imaging

To monitor and quantify the effects of resveratrol incubation on IMCL accumulation in vitro over time, we first performed live cell imaging in myotubes from an untrained donor for 48 h with 1‐h intervals. Live cell imaging was performed using a FEI Corrsight spinning disk confocal microscope, equipped with an ORCA‐Flash 4.0 V2 CMOS camera, using a 40× 0.9 N.A. air objective (Zeiss) at 37°C. Bodipy 493/503 (D3922; Molecular probes, Fisher Scientific; 1:250) was added to the medium (and remained present throughout the imaging period) to visualize the LDs and CellMask (C10046; Invitrogen, Fisher Scientific; 1:1000) was used to visualize the plasma membranes. Upon thresholding the Bodipy‐derived signal, total lipid area, total LD number and average LD size were quantified in multiple wells.

### 2.6 Visualization of fixed cells

Based upon the results from live cell imaging, we picked the 48‐h time point for detailed examination in fixed cells. Human primary myotubes from three different donors were grown on #1 coverslips and incubated with resveratrol for 48 h. Myotubes were fixed with 3.7% formaldehyde and stained with 286 nM DAPI (4’,6‐diamidino‐2‐phenylindole, dihydrochloride; D1306; Invitrogen) to visualize the nuclei and stained with Bodipy 493/503 (1:100) to visualize the LDs. Coverslips were mounted on glass slides with Mowiol. Imaging was performed with a FEI Corrsight spinning‐disk confocal microscope, using a 63× 1.4 N.A. oil immersion objective (Zeiss) and with a Nikon E800 fluorescence microscope (Nikon), coupled to a Nikon DS‐Fi1c colour CCD camera (Nikon), using a 40x objective. Images obtained from the Corrsight were analysed similar to the live cell images. Images from the Nikon microscope were analysed upon thresholding and Bodipy‐derived signal was corrected for the number of nuclei.

### 2.7 High‐resolution confocal imaging of lipid droplet characteristics

#### 2.7.1 Immunohistochemistry

Biopsies from the m. *vastus lateralis* were frozen in liquid nitrogen‐cooled isopentane. Five‐µm‐thick sections were serial cut with a cryostat (CM3050; Leica Biosystems) at −20°C, mounted on glass slides and air dried for 30 min. To ensure identical incubation conditions, sections from placebo and resveratrol were mounted on the same glass slide. Slides were fixed with 3.7% formaldehyde and permeabilized with 0.25% TX‐100. Primary antibodies against Laminin (L9393; Sigma‐Aldrich; 1:25), MHC‐1 (A4.840; Developmental Studies Hybridoma Bank; 1:25) and PLIN5 (GP31; ProGen Biotechnik; 1:25) were used. Appropriate secondary antibodies were conjugated with AlexaFluor (AF) 405, AF555 and AF647. Bodipy 493/503 (1:100) was applied simultaneously with the secondary antibodies to visualize LDs.

#### 2.7.2 Imaging and quantification

Imaging and quantification of IMCL, LD size, number and subcellular distribution in type I and type II muscle fibres was essentially performed according to Daemen et al. (2018). Briefly, cross‐sections of circa 20 muscle fibres (with a fibre type I/II ratio that matched the individual fibre typing (on average 45% type I and 55% type II fibres)) were imaged with a Leica TCS SP8 confocal microscope using a 63 × 1.4 N.A. oil immersion objective. Deconvolution was performed with Huygens Professional software (Scientific Volume Imaging B.V.), quantification by ImageJ version 1.52p (NIH, Bethesda, USA) (Schneider et al., 2012) and further calculations with Matlab R2013a (The Mathworks, Inc.). IMCL was determined as the total lipid area corrected for the total cell area. LD number is defined as total LD count corrected for cell area and LD size is the total lipid area divided by the total LD count. Subcellular distribution was analysed by making a distinction between intermyofibrillar (IMF) LDs or subsarcolemmal (SS) LDs, whereas the SS region represents the outer 8% of each cell and the IMF region the remaining 92% of the cell. Moreover, LDs were subdivided into PLIN5‐positive LDs (PLIN5+) and LDs without PLIN5 (PLIN5‐), as previously described (Gemmink et al., 2018).

### 2.8 Western blot

Protein levels of PLIN5 in human muscle tissue were determined by Western blot in 8/10 individuals from the subgroup. Tissues were lysed using Bio‐Plex Cell Lysis kit (171‐304011; Bio‐Rad) and equal amounts of protein were loaded onto the gels (Figure S1). After gel electrophoresis, proteins were transferred by Western blotting and a Revert total protein stain (LI‐COR Bioscience, Westburg) was performed to determine the protein quality and the protein quantity on the blots. Then, the membranes were blocked with LI‐COR Blocking buffer for 30 min and incubated with a primary antibody against PLIN5 (1:2500, diluted in LI‐COR Blocking buffer) overnight at room temperature. A IRDye800‐conjugated secondary antibody was used for visualization PLIN5 by an Odyssey near infrared scanner (LI‐COR Biosciences). Western blots were quantified with Image Studio version 5.2 (LI‐COR Biosciences) and values were normalized to total protein stain.

### 2.9 Statistical analysis

Statistical analysis was performed with Prism version 8.2 (GraphPad) and SPSS, version 25 (IBM Corp.). After testing for normality (Shapiro–Wilk normality test), differences between placebo and resveratrol were tested by two‐tailed Student's paired *t*‐test. Time course analysis was performed by two‐way ANOVA. Results are presented as mean ± SEM, unless indicated otherwise. A *p*‐value <0.05 was considered statistically significant.

## 3 RESULTS

### 3.1 Resveratrol stimulates lipid accumulation in a cell autonomous manner

**FIGURE 1:**
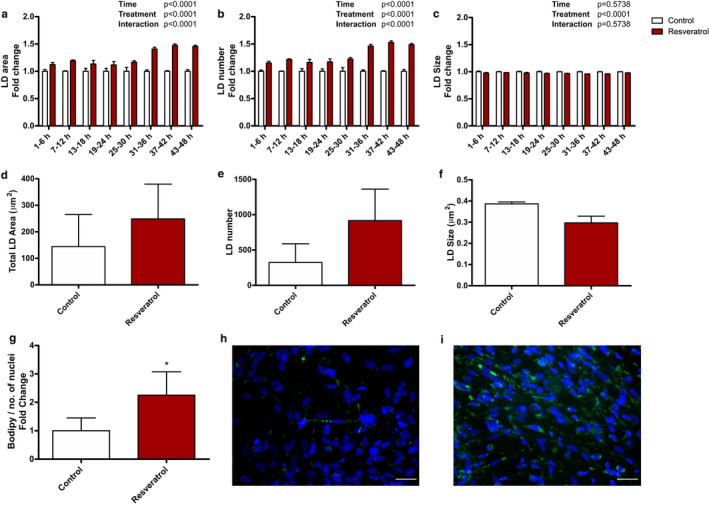
Resveratrol treatment stimulates lipid accumulation in human muscle cells. Human myoblasts were cultured and differentiated and incubated with or without 50 ng/ml resveratrol. (a–c) Time course analysis for LD area (a), LD number (b) and LD size (c). Data are obtained from one donor, from multiple wells. Data are presented as mean fold change ± SEM (*n* = 6 time points); analysed by two‐way ANOVA. (d–f) Effect of resveratrol on total LD area (d), total LD number (e) and average LD size (f) after 48 h incubation. Data are obtained from three donors, from multiple wells in fixed cells. Data are presented as mean ±SEM (*n* = 3). (g–i) After 48 h incubation, cells were fixed and nuclei were stained with DAPI (blue) and neutral lipids with Bodipy 493/503 (green). Bodipy area/nuclei was determined with or without resveratrol. Data are obtained from three donors and 2–4 repeated experiments. Data are presented as mean fold change ± SEM (*n* = 9). *Significantly different from control (*p* < 0.05, paired *t*‐test). Images are representative for (g) incubated without (h) and with resveratrol (i). Scale bar = 40 µm

Resveratrol has previously been shown to augment IMCL in vivo (Table 1) (de Ligt et al., 2018; Timmers et al., 2011, 2016). To examine if these effects were muscle cell autonomous, we incubated human primary muscle cells with resveratrol and used fluorescent microscopy to determine intracellular lipid accumulation. First, lipid accumulation was followed by live cell imaging after resveratrol incubation, revealing an overall significant time and treatment effect. Within the first 6 h, LD area was 12% higher after resveratrol treatment compared to control. This difference between the conditions was sustained and increased towards ~41–46% from 31 to 48 h (Figure 1a). This increase in LD area originated from an increase in LD number (Figure 1b), with a 15% greater LD number within the first 6 h upon resveratrol treatment, which further increased to 46–49% between 31 and 48 h compared to control. No changes in LD size were observed (Figure 1c). Subsequently, we chose the 48‐h time point to perform cell experiments with myotubes from three different untrained donors that were incubated with resveratrol. In fixed cells, lipid area increased after 48 h (1.7‐fold increase, Figure 1d). This increase originates predominantly from an increase in LD number (placebo 323 ± 265 vs. resveratrol 917 ± 445, Figure 1e) rather than size (placebo 0.39 ± 0.01 µm^2^ vs. resveratrol 0.30 ± 0.03 µm^2^, Figure 1f). Moreover, LD accumulation was related to the number of nuclei to obtain values related to cell number and promote comparison to the in vivo analyses. From multiple experiments, we observed a 2.3‐fold increase (*p* = 0.038, Figure 1g) in lipid area relative to nuclei (placebo vs. resveratrol, Figure 1h vs. 1i).

Resveratrol supplementation in vivo induces LD accumulation in a fibre type and subcellular region‐specific fashion.

**FIGURE 2:**
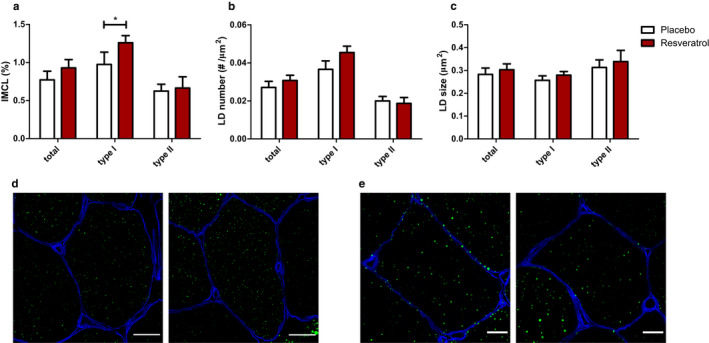
Resveratrol increases IMCL in type I muscle fibres. Effect of in vivo resveratrol supplementation of 150 mg/day trans‐resveratrol on IMCL (a), LD number (b) and LD size (c) in human muscle biopsies. IMCL was determined by lipid area (Bodipy staining)/cell area * 100%. Parameters were analysed in type I muscle fibres, type II muscle fibres and for both fibre types combined. Data are presented as mean ± SEM (*n* = 10); *p < 0.05 (paired *t*‐test). Images are representative for IMCL in type I muscle fibres (d) and type II (e) muscle fibres with placebo on the left and resveratrol on the right. Cell membranes are stained in blue and LDs in green. Scale bar is 20 µm (d) or 10 µm (e)

In metabolically compromised individuals, 30 days of resveratrol supplementation augmented IMCL content (Table 1). Detailed analysis revealed that IMCL increased in type I muscle fibres (placebo 0.97 ± 0.16% vs. resveratrol 1.26 ± 0.09%; *p* = 0.030; Figure 2a and d) but not in type II muscle fibres (placebo 0.62 ± 0.09% vs. resveratrol 0.66 ± 0.15%; *p* = 0.794; Figure 2a and e). While in the cell model, resveratrol‐induced increase in LD originated predominantly from an increase in LD number, the significant increase in total IMCL in vivo originates from the combined effect of a non‐significant increase in LD number (placebo 0.037 ± 0.004 µm^−2^ vs. resveratrol 0.045 ± 0.003 µm^−2^; *p* = 0.083; Figure 2b) and a non‐significant increase in size (placebo 0.26 ± 0.02 µm^2^ vs. resveratrol 0.28 ± 0.02 µm^2^; *p* = 0.181; Figure 2c).

**FIGURE 3:**
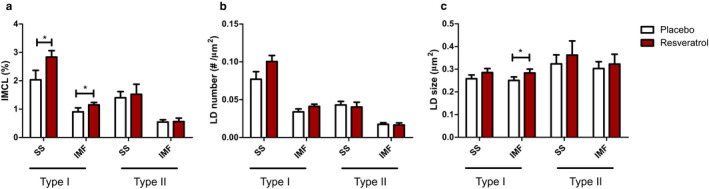
Resveratrol increases IMCL in type I muscle fibres in both the intermyofibrillar (IMF) and subsarcolemmal (SS) region. Effect of in vivo resveratrol supplementation of 150 mg/day trans‐resveratrol on IMCL (a), lipid droplet (LD) number (b) and LD size (c) in human muscle biopsies. LDs are analysed separately in the IMF region and SS region, in both type I and type II muscle fibres. IMCL was determined by lipid area (Bodipy staining)/cell area*100%. Data are presented as mean ± SEM (*n* = 10); *p < 0.05 (paired *t*‐test)

Examination of subcellular distribution of LDs in type I fibres revealed an increase in IMCL in the SS as well as in the intermyofibrillar IMF region (2.04 ± 0.33% vs. 2.83 ± 0.23%, *p* = 0.016 for placebo vs. resveratrol in the SS region and 0.90 ± 0.14% vs. 1.15 ± 0.08%, *p* = 0.026 for placebo vs. resveratrol in the IMF region; Figure 3a). While in the SS region, the increase in total LD content was caused by a non‐significant increase in LD number (placebo 0.077 ± 0.010 µm^−2^ vs. resveratrol 0.100 ± 0.008 µm^−2^; *p* = 0.069, Figure 3b) and LD size (placebo 0.26 ± 0.02 µm^2^ vs. resveratrol 0.29 ± 0.02 µm^2^; *p* = 0.080, Figure 3c), the change in LD content in the IMF region originated from a non‐significant increase in LD number (placebo 0.034 ± 0.004 µm^−2^ vs. resveratrol 0.041 ± 0.003 µm^−2^; *p* = 0.104, Figure 3b) and a significant increase in LD size (placebo 0.25 ± 0.02 µm^2^ vs. resveratrol 0.28 ± 0.02 µm^2^; *p* = 0.042, Figure 3c). No significant changes were observed in type II muscle fibres for IMCL (SS: *p* = 0.692; IMF: *p* = 0.881), LD number (SS: *p* = 0.757; IMF: *p* = 0.813) and LD size (SS: *p* = 0.262; IMF: *p* = 0.452) (Figure 3a–c).

### 3.2 Resveratrol promotes IMCL storage predominantly in LDs coated with PLIN5 present in type I muscle fibres

**FIGURE 4:**
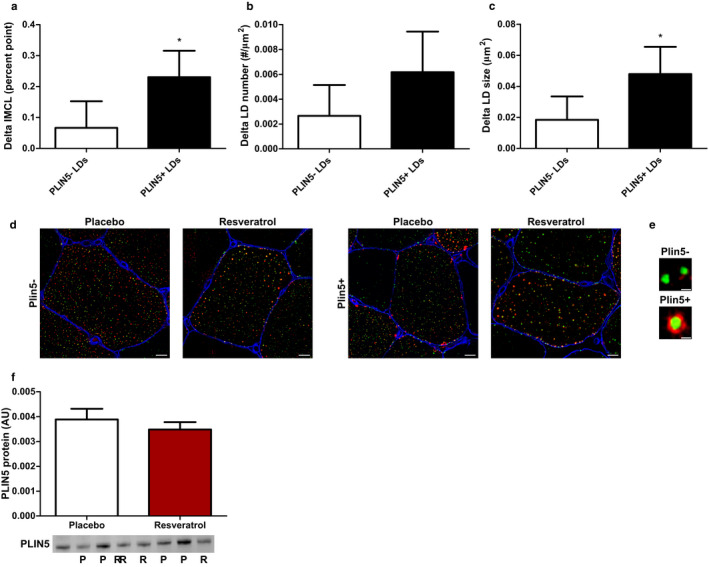
Resveratrol increases IMCL in specifically PLIN5‐coated lipid droplets in type I muscle fibres. LDs are distinguished between PLIN5‐negative LDs (PLIN5‐) and PLIN5‐positive LDs (PLIN5+). The increase in IMCL after supplementation of 150 mg/day trans‐resveratrol was significant in PLIN5+ LDs and not in PLIN5‐ LDs (a). This increase in IMCL was caused by a non‐significant increase in LD number (b) and to a significant increase in LD size (c). Data are from human muscle biopsies and presented as mean delta ± SEM (*n* = 10); *significant difference between placebo and resveratrol (*p* < 0.05; paired *t*‐test). The increase in IMCL after resveratrol supplementation is visualized in representative images for PLIN5‐ LDs and PLIN5+ LDs. Scale bar = 10 µm (d). A LD is PLIN5+ when there is overlay of PLIN5 staining with the LD staining. Scale bar = 1 µm (e). Total PLIN5 protein did not change after resveratrol supplementation determined via Western blot. Blot images are from four different individuals. P = placebo; R = resveratrol. Data are from human muscle biopsies and presented as mean ± SEM (*n* = 8).

We and others previously observed that preferential storage of lipids in LDs coated with PLIN5 permits augmented IMCL content in oxidative muscle, while not impeding insulin sensitivity (Gemmink et al., 2016, 2018; Shepherd et al., 2017). To examine the preferred lipid storage in LDs coated with PLIN5 in the resveratrol‐mediated increase in lipid content in oxidative muscle, we made the distinction between LDs coated with PLIN5 (PLIN5+ LDs) and LDs without PLIN5 (PLIN5‐ LDs) (Figure 4e). Interestingly, we observed a significant increase in lipid storage in PLIN5+ LDs (delta increase 0.231 per cent point, *p* = 0.024, Figure 4a+d) but not in PLIN5‐ LDs (delta increase 0.067 per cent point, *p* = 0.457, Figure 4a+d) in type I muscle fibres upon resveratrol supplementation. The increase in lipid storage in PLIN5+ LDs was accounted for by a non‐significant increased number of PLIN5+ LDs (*p* = 0.091, Figure 4b) and a significant increase in size (*p* = 0.023; Figure 4c). For type II muscle fibres (known to have limited PLIN5 content), no such observations were made (IMCL type II fibres PLIN5+ LDs; delta increase −0.022 per cent point, *p* = 0.466). PLIN5 protein content was not different between placebo and resveratrol (Figure 4F).

## 4 DISCUSSION

The current study showed that resveratrol augments IMCL content in human skeletal muscle and results in remodelling of LDs in a cell autonomous fashion. In human clinical trials, it has been observed that 30 days of 150 mg/day resveratrol supplementation can augment overall IMCL storage without impeding insulin sensitivity in metabolically compromised individuals. Hypothetically, this is indicative of a benign ‘athlete‐like’ IMCL storage pattern. In line with this hypothesis, we observed that resveratrol particularly promoted IMCL storage in oxidative type I muscle fibres and LDs coated with PLIN5.

The cell model indicates that the IMCL inducing effects of resveratrol are not secondary to alterations in whole‐body lipid metabolism and the increase in IMCL upon resveratrol originated from an increase in LD number. In the human intervention study, resveratrol also induced a significant increase in overall IMCL content. The increase in IMCL originates from the combined effect of a 24% non‐significant (*p* = 0.083) increase in LD number without profound changes in LD size. The direction of these morphological changes indeed matches with an athlete like phenotype (Gemmink et al., 2020). Unlike in athletes, the resveratrol‐mediated increase in IMCL was not only found in intermyofibrillar region but in the subsarcolemmal region as well.

Type I muscle fibres are known to store higher amounts of lipids compared to type II muscle fibres and are more oxidative (Schrauwen‐Hinderling et al., 2006; van Loon et al., 2003, 2004). For storage of IMCL in type I fibres, we previously reported that the number of LDs correlates positively with insulin sensitivity, whereas in type II fibres the size of LDs correlates negatively with insulin sensitivity (Daemen et al., 2018). Thus, the preferred storage of IMCL in type I fibres rather than in type II fibres and in more LDs rather than large LDs matches with the observation that the resveratrol‐mediated increase in IMCL does not impede insulin sensitivity.

Interestingly, the increase in IMCL observed in type I muscle fibres was most profound in LDs coated with PLIN5. Preferred storage of IMCL in PLIN5+ LDs was previously observed in trained athletes (Gemmink et al., 2018) and found to ameliorate insulin resistance (Gemmink et al., 2016). Again, this fits the hypothesis that resveratrol promotes lipid storage in a benign, athlete‐like fashion. PLIN5 is a LD coat protein with a major role in lipid turnover (MacPherson et al., 2013; Wang, Bell, et al., 2011) and supports to match LD lipolysis with mitochondrial fat oxidation to prevent lipotoxicity and insulin resistance (Bosma et al., 2013; Laurens et al., 2016; Wang, Sreenevasan, et al., 2011). To our knowledge, no research has currently been performed to investigate the role of resveratrol on PLIN5 in humans. In mice, resveratrol was reported to increase PLIN5 protein content in the muscle (Mehdi et al., 2018). In the present human study, we did not find an increase in total PLIN5 content. In fact, the observed increase in PLIN5+ LDs (in the absence of changes in total PLIN5 content) is suggestive of PLIN5 redistribution (Gemmink et al., 2016; Shepherd et al., 2017). The current study design did not permit more mechanistic studies as to how resveratrol may mediate augmented lipid storage in PLIN5‐coated LDs in type I fibres. While pre‐clinical models revealed activation of PGC1α upon resveratrol (Lagouge et al., 2006), this is not observed consistently (Higashida et al., 2013). Although the results on induction of PGC1α in human skeletal muscle by resveratrol are equivocal, it is important to note that overexpression of PGC1α in skeletal muscle induces gene and protein expression of a series of proteins involved in LD dynamics including PLIN5 (Koves et al., 2013).

In contrast to observations in trained athletes, the resveratrol‐mediated increase in IMCL was not limited to the IMF region, but was also observed in the SS region. High levels of SS LDs are associated with lower insulin sensitivity (Chee et al., 2016; Daemen et al., 2018; Nielsen et al., 2010). Fibre type‐specific examinations revealed that IMCL stored in SS LDs impeded insulin sensitivity particularly if found in type II and not type I fibres (Daemen et al., 2018). The induction of IMCL in SS LDs upon resveratrol was almost exclusively observed in type I fibres, possibly explaining why this increase was not paralleled by compromised insulin sensitivity. Why resveratrol promotes lipid storage in both the SS and in the IMF compartments is currently not known. Based on anatomical location, one could speculate that the vicinity of SS LDs with the capillary network encompassing the type I fibres renders SS LDs, a physically nearby storage site for extracted circulatory fats. On the other hand, IMF LDs are closely located to the contractile elements (Tarnopolsky et al., 2007) and have been suggested to fuel mitochondrial fat oxidation during prolonged submaximal exercise (Hood, 2001). Upon exercise or exercise training, fatty acids can be released from SS LD’s and be re‐incorporated in IMF LDs to fuel mitochondrial oxidation upon demand. The observation that lipid content in SS LDs decreases after exercise without changes in lipid content in IMF LDs support this notion (Li et al., 2014; Nielsen et al., 2010). Regular physical exercise is an obvious difference between changes in the athletes versus the resveratrol‐mediated increase in IMCL content in a metabolically compromised non‐physically active population observed here. Regular physical exercise requires an efficient system for IMF LD turnover. Administration of resveratrol in the absence of regular exercise may not trigger such a mechanism, which may explain the increase in both IMF and SS LDs.

This is the first study that has examined the effect of resveratrol on intramyocellular lipid storage in primary human muscle cells as well as in vivo in humans. Our unique quantitative microscopy approach permits examination of the effect of resveratrol in a fibre type and region‐specific fashion at the level of individual lipid droplets. More conventional approaches will only give results based on muscle homogenates or extractions that will not result in the type of detailed information needed to explain how resveratrol can augment IMCL content while not impeding insulin sensitivity. The downside of this approach is that it is extremely laborious, which limits the number of subjects that could be included in the current study.

In conclusion, we observed that resveratrol stimulates lipid accumulation in human muscle cells in culture as well as in vivo in metabolically compromised humans. The pattern of IMCL storage upon administration of resveratrol mimics the pattern of IMCL storage in trained athletes. Deduced from this resemblance, a phenotype of benign IMCL storage is emerging with LDs being stored in type I muscle fibres in numerous small LDs most of which are coated with PLIN5. Thus, a favourable remodelling of the IMCL storage pattern can be achieved upon resveratrol administration.

## Supporting information



Figure S1
