# Supplementary material for: Resveratrol‐induced remodelling of myocellular lipid stores: A study in metabolically compromised humans
Source: Physiol Rep. 2021 Jan 21;9(2):e14692. doi: 10.14814/phy2.14692 (PMC7819107; doi:10.14814/phy2.14692)
Supplement: Supplementary file 1 — Figure S1 [file PHY2-9-e14692-s001.docx]

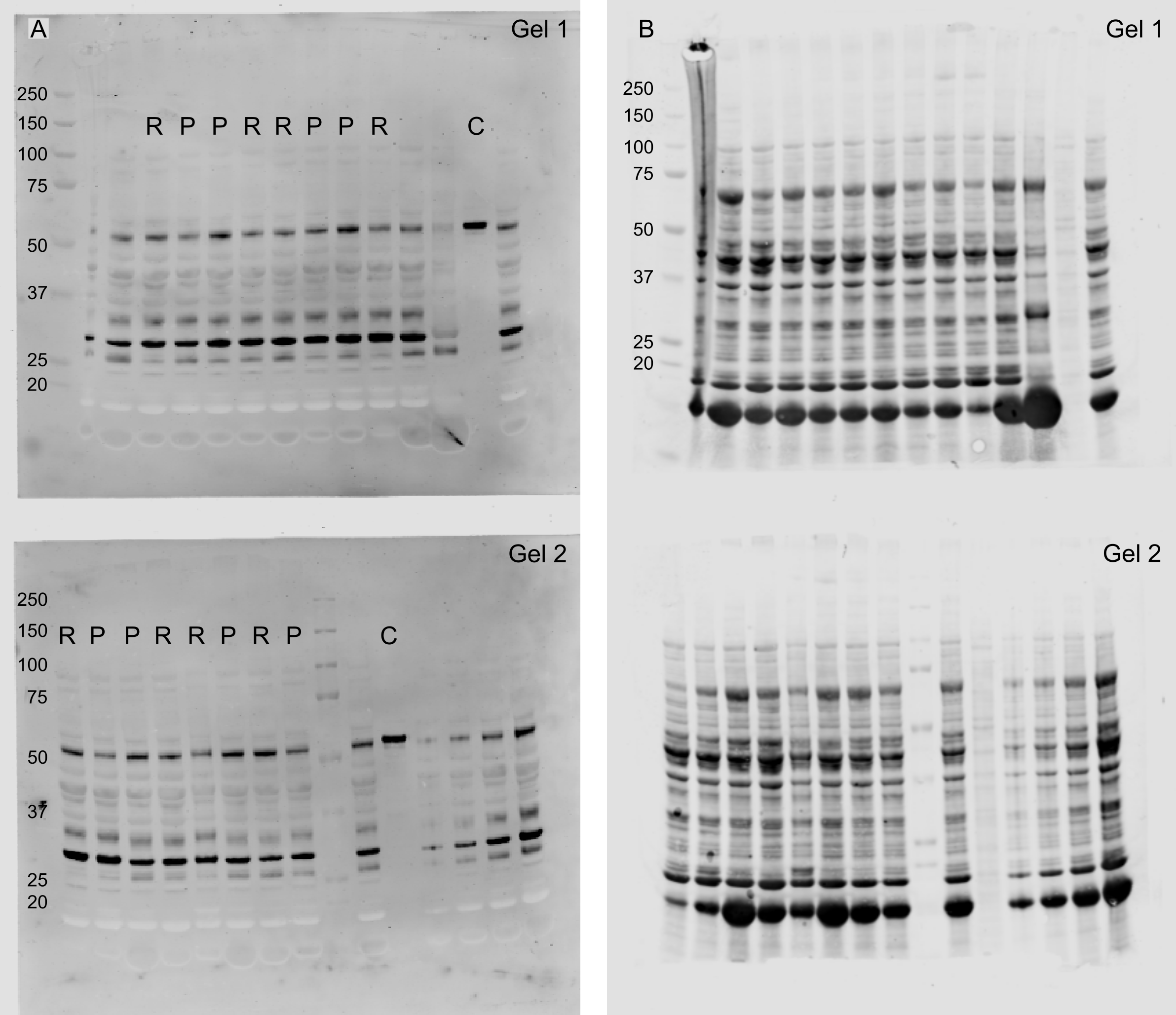


***Figure S1. PLIN5 western blot****. Images of the PLIN5 western blot (A) and Revert total protein stain (B). In eight individuals, PLIN5 protein was analyzed after placebo (P) and resveratrol (R) supplementation, where 4 paired samples were run on each gel (A). Samples were compared with a positive control (C). The numbers of the protein ladders are in kD and present in lane 1 on gel 1 and in lane 9 on gel 2.*
